# Thymus, undernutrition, and infection: Approaching cellular and molecular interactions

**DOI:** 10.3389/fnut.2022.948488

**Published:** 2022-09-26

**Authors:** Wilson Savino, Jonathan Durães, Carolina Maldonado-Galdeano, Gabriela Perdigon, Daniella Arêas Mendes-da-Cruz, Patricia Cuervo

**Affiliations:** ^1^Laboratory on Thymus Research, Oswaldo Cruz Institute, Oswaldo Cruz Foundation, Rio de Janeiro, Brazil; ^2^Brazilian National Institute of Science and Technology on Neuroimmunomodulation, Oswaldo Cruz Institute, Oswaldo Cruz Foundation, Rio de Janeiro, Brazil; ^3^Rio de Janeiro Research Network on Neuroinflammation, Oswaldo Cruz Institute, Oswaldo Cruz Foundation, Rio de Janeiro, Brazil; ^4^Laboratory on Leishmaniasis Research, Oswaldo Cruz Institute, Oswaldo Cruz Foundation, Rio de Janeiro, Brazil; ^5^Laboratory of Immunology, Reference Center for Lactobacilli Centro de Referencia para Lactobacilos-Consejo Nacional de Investigaciones Científicas y Técnicas (CERELA-CONICET), San Miguel de Tucumán, Argentina; ^6^Laboratory of Immunology, Faculty of Biochemistry, Chemistry and Pharmacy, National University of Tucumán, San Miguel de Tucumán, Argentina; ^7^School of Pharmacy and Biomedical Sciences, University of Central Lancashire, Preston, United Kingdom

**Keywords:** undernutrition, thymus, infectious diseases, visceral leishmaniasis, Chagas disease, probiotics

## Abstract

Undernutrition remains a major issue in global health. Low protein-energy consumption, results in stunting, wasting and/or underweight, three deleterious forms of malnutrition that affect roughly 200 million children under the age of five years. Undernutrition compromises the immune system with the generation of various degrees of immunodeficiency, which in turn, renders undernourished individuals more sensitive to acute infections. The severity of various infectious diseases including visceral leishmaniasis (VL), influenza, and tuberculosis is associated with undernutrition. Immunosuppression resulting from protein-energy undernutrition severely impacts primary and secondary lymphoid organs involved in the response to related pathogens. The thymus—a primary lymphoid organ responsible for the generation of T lymphocytes—is particularly compromised by both undernutrition and infectious diseases. In this respect, we will discuss herein various intrathymic cellular and molecular interactions seen in undernutrition alone or in combination with acute infections. Many examples illustrated in studies on humans and experimental animals clearly revealed that protein-related undernutrition causes thymic atrophy, with cortical thymocyte depletion. Moreover, the non-lymphoid microenvironmental compartment of the organ undergoes important changes in thymic epithelial cells, including their secretory products such as hormones and extracellular matrix proteins. Of note, deficiencies in vitamins and trace elements also induce thymic atrophy. Interestingly, among the molecular interactions involved in the control of undernutrition-induced thymic atrophy is a hormonal imbalance with a rise in glucocorticoids and a decrease in leptin serum levels. Undernutrition also yields a negative impact of acute infections upon the thymus, frequently with the intrathymic detection of pathogens or their antigens. For instance, undernourished mice infected with *Leishmania infantum* (that causes VL) undergo drastic thymic atrophy, with significant reduction in thymocyte numbers, and decreased levels of intrathymic chemokines and cytokines, indicating that both lymphoid and microenvironmental compartments of the organ are affected. Lastly, recent data revealed that some probiotic bacteria or probiotic fermented milks improve the thymus status in a model of malnutrition, thus raising a new field for investigation, namely the thymus-gut connection, indicating that probiotics can be envisioned as a further adjuvant therapy in the control of thymic changes in undernutrition accompanied or not by infection.

## Introduction

Despite one of the global health priorities listed in the Sustainable Development Goals (SDGs) by the United Nations is combating hunger and ensuring sustainable food security and proper nutrition (1), malnutrition remains a serious public health problem worldwide. The World Health Organization (WHO) defines malnutrition as the deficiency or excess in the consumption of specific energy and/or nutrients in relation to the needs of an individual, resulting in corresponding pathologies: undernutrition or obesity (2). The lack of macro and micronutrients, and in particular the low consumption of proteins and calories results in four deleterious forms of undernutrition: (i) wasting, (ii) stunting, (iii) underweight, and (iv) micronutrient deficiencies. (i) Wasting is characterized as low weight-for-height, and in children it may be lethal if not appropriately treated; (ii) stunting occurs when the individual presents low height for the respective age, usually due to chronic undernutrition; (iii) underweight is defined as low weight-for-age, and underweighted children may be stunted, wasted or both. Lastly, (iv) micronutrient undernutrition refers to lack of vitamins and minerals that are essential for body functions (2). It is estimated that stunt and wasting affect virtually 200 million children under 5 years of age around the world, whereas in the adult population this number can reach 462 million. On the other hand, around 42 million children globally are overweight or obese (2).

Malnutrition thus constitutes a serious global public health issue, particularly in developing countries. According to a recent report (3) of the Food and Agriculture Organization (FAO) of the United Nations, 720–811 million people in the world suffered from hunger in 2020, increasing by 161 million the number of people who experienced hunger in 2019. The COVID-19 pandemic contributed substantially to this increase. The global assessment of food insecurity and malnutrition for 2020 shows that not only world hunger has increased, but also the prevalence of undernutrition rose by 4% in one single year (3).

**FIGURE 1:**
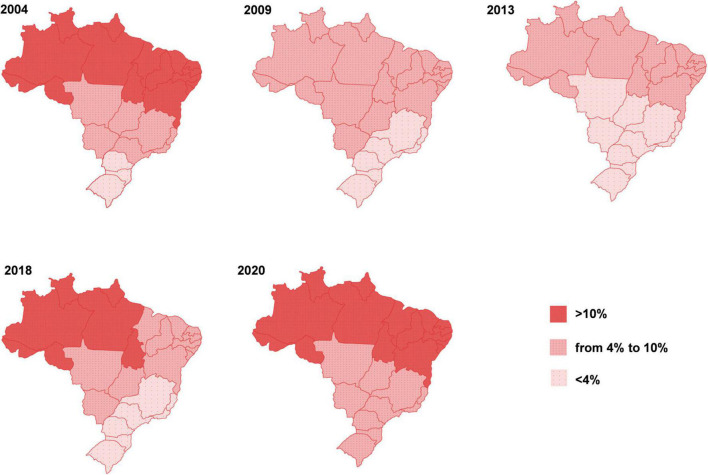
Evolution of hunger in Brazil. The figure shows the percentage of the population affected by severe food insecurity from 2004 to 2020, seen in different macro-regions of the country. Figure reproduced (with permission) from the Report of the National Survey of Food Insecurity in the Context the COVID-19 Pandemic in Brazil published by the Brazilian Research Network of Food and Nutrition Sovereignty and Security (applied herein with permission from Rede PENSSAN, http://olheparaafome.com.br/VIGISAN_Inseguranca_alimentar.pdf).

Due to the pandemic, the prevalence of different forms of malnutrition has increased worldwide and it is estimated that these effects will be lasting, as already seen in 2021. In fact, the COVID-19 pandemic has widened and worsened inequalities between countries, affecting the livelihoods of an estimated 1.6 billion workers in the formal economy (1). Around 12% of the world’s population suffered from severe food insecurity in 2020. In Latin America and Caribbean region in 2020, 14 million more people were affected by hunger as compared with 2019 (3). Data from the National Survey on Food Insecurity in the Context of the COVID-19 Pandemic in Brazil showed that the country went back 15 years in five, and hunger returned to be a structural problem (4), as depicted in Figure 1.

The decrease in proper food intake results in a series of physiological changes, including: (i) growth restriction; (ii) reduction of fat, muscle, and visceral mass; as well as (iii) reduction in basal metabolic rate and energy expenditure (5). These alterations also comprise biochemical parameters with reduced levels of triiodothyroxine (T3), insulin, insulin-like growth factor-1 (IGF-1) and leptin, among others; together with increased levels of growth hormone (GH) and cortisol (5, 6). Overall, undernutrition induces changes in metabolic, hormonal and glucoregulatory mechanisms (5, 6). Also, undernutrition modulates the intestinal microbiota and dysbiotic events can be observed as a cause and/or consequence of undernutrition, accompanied by local and systemic chronic inflammation (7). Altered nutrient absorption and chronic inflammation seems to be related to the fact that malnourished individuals are more susceptible to various diseases (7–9).

The dynamic relationship between infection, nutrition and immunity is long recognized. Being a systemic disease, undernutrition also affects primary and secondary lymphoid organs, harming the immune system of malnourished individuals (10). Since the immune response depends on cell replication and the synthesis of active protein compounds, undernutrition clearly has a negative impact on immunity (11). Actually, immunosuppression caused by protein-energy undernutrition increases susceptibility to acute infections and leads to the development of more severe forms of disease, whether they are caused by parasites, protozoa, bacteria or viruses.

In the present review we aimed at focusing the effects of undernutrition and infection upon one specific compartment of the immune system, namely the thymus, particularly the changes in the organ involving cellular and molecular interactions. Yet, before going into this point, it seems worthwhile to provide a general, yet concise background of the thymus and the generation of T-lymphocytes.

## The thymic microenvironment and intrathymic T-cell differentiation

The thymus is a primary organ in the immune response, where the maturation and differentiation of T cells take place (12). The correct selection and migration of T cells occurs through a series of proliferation and differentiation stages dependent upon receiving instructions from the specialized thymus non-lymphoid microenvironment (13). In mammals, the organ is histologically divided lobules, each one comprising two main regions, namely cortex and medulla, with the cortex being denser in lymphocytes than the medulla. Developing thymocytes in different stages of maturation are specifically located in those regions. For example, immature CD4-CD8- double-negative (DN) and CD4^+^ CD8^+^ double-positive (DP) thymocytes are localized in the cortical region of the thymic lobules, whereas more mature CD4^+^ CD8- or CD4-CD8^+^ single-positive (SP) thymocytes are placed in the medulla (13). Such specific distribution indicates that thymocyte maturation occurs in parallel with organized and coordinated cell migration (14). In fact, disruption or abnormal cell migration impacts thymocyte development, as seen for example in Chagas disease (15).

Intrathymic thymocyte differentiation and migration, from the entrance of precursor cells to the exit of mature SP cells, is dependent on interactions controlled by the thymic tridimensional network. This is composed by cellular components such as thymic epithelial cells (TEC), thymic dendritic cells (TDC), macrophages and fibroblasts, as well as non-soluble and soluble molecules such as the extracellular matrix (ECM) proteins fibronectin, laminin, type I and IV collagens; cytokines as interleukin (IL)-2, IL-6, IL-7, and IL-22; chemokines as CXCL12, CCL4, and CCL7; hormones as thymosin, thymopoietin, and thymulin; and different typical soluble components of nervous tissues, such as neuropeptides and neurotransmitters (12, 15, 16).

During thymocyte development, cells pass through the positive selection and negative selection events; both being derived from the molecular interactions involving expression and survival signaling by the recognition of the T-cell receptor (TCR), upon interacting with microenvironmental cells, particularly dendritic cells, and epithelial cells. Positive selection can be defined as the process through which TCR-expressing CD4^+^ CD8^+^ developing thymocytes are rescued from programmed cell death upon interaction with self-antigens presented to the TCR by molecules of the major histocompatibility complex (MHC) by thymic microenvironmental cells. Positively selected thymocytes still in the cortex of the thymic lobules then move into the boundary of the cortex and medulla of the thymus for the presentation of self-antigens by the medullary thymic epithelial cells for the second time. In the medulla, thymocytes pass through the negative selection. In this case, differentiating cells undergo apoptosis if their TCR interact with high avidity with self-antigens coupled to the MHC class I or class II molecules expressed by microenvironmental cells in the organ (12, 13). Different peripheral tissue antigens (PTAs)—or self-antigens—coupled to MHC molecules are expressed on the membrane of medullary thymic epithelial cells (mTEC). Such expression is regulated by the autoimmune regulator (AIRE) transcription factor and avoids the development of self-antigen reactive cells, therefore preventing autoimmunity (17, 18). Alternatively, some clones that recognize self-antigens with high avidity become regulatory CD4^+^ CD25^+^ Foxp3^+^ T cells (Treg), through a mechanism dependent on the TCR signaling avidity and duration (19, 20).

Positioning of developing thymocytes along with differentiation depend on multiple interactions involving cell-cell, cell-matrix as well as chemokine-mediated interactions. For example, CXCL12 is secreted by TEC, and preferentially attracts immature CD4-CD8- and CD4^+^ CD8^+^ cells, by ligation with the receptor CXCR4. The chemokine CCL25 also attracts immature thymocytes, although all thymocyte subsets are responsive, which is in keeping with the fact that its CCR9 receptor is expressed at all stages of murine thymocyte differentiation. Interestingly, CCL19/CCR7 interactions participate in thymocyte exit, illustrating that thymocytes may switch their responses to a given chemokine, tuning their migratory process (21).

Other *stimuli* can participate in the overall process of intrathymic T-cell development, including hormones. Thymulin is zinc-couple nonapeptide classically defined as a thymic hormone, being produced by TEC. This molecule enhances developing thymocyte proliferation and IL-2 production (22). Interestingly, TECs also produce other thymic hormones as thymopoietin and thymosin*-*α1 (23), both known to have extrathymic functions, but also to induce progression of thymocyte differentiation.

**FIGURE 2:**
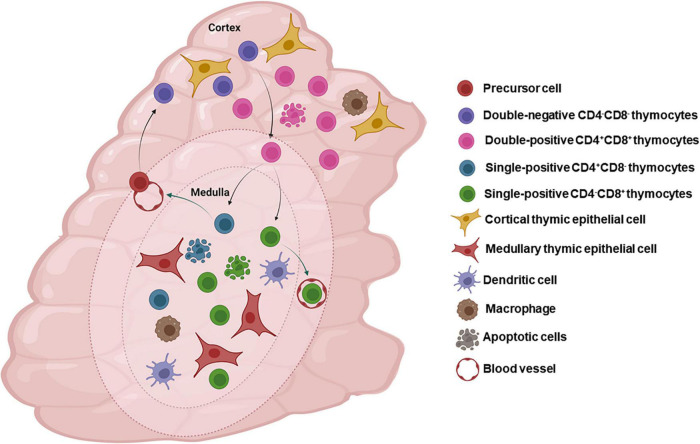
The thymic microenvironment and normal intrathymic T-cell differentiation. The panel schematically depicts in a concise way, the general process of thymocyte differentiation, in the context of the thymic microenvironment. Bone marrow-derived precursors enter the organ through blood vessels in the corticomedullary junction and migrate toward the outer cortex where they proliferate, but do not express the CD3/TCR complexes as well as the accessory molecules CD4 and CD8. There CD4/CD8 double-negative cells (DN) evolve to express TCR and become CD4^+^CD8^+^ (double-positive cells) and, under the control of the thymic microenvironment, undergo positive selection, with positively selected thymocytes migrating toward the medulla, where a large majority will die by negative selection, though apoptosis, and are ultimately resorbed within the organ by macrophages. Mature CD4^+^ or CD8^+^ single-positive thymocytes will eventually leave the organ to colonize the T-cell regions of peripheral lymphoid organs. Most thymocytes interact with microenvironmental cells in the cortical and medullary regions of the thymic lobules. Figure created with BioRender.com.

Overall, we can say that in physiological conditions, at the end of their journey, CD4-SP and CD8-SP thymocytes exit the thymus to populate the peripheral lymphoid organs and participate in adaptive immune responses. The main stages of thymocyte maturation, selective processes and components of the thymic microenvironment are represented in Figure 2.

## Undernutrition causes thymic atrophy, with cortical thymocyte depletion

It is well described that the intestinal homeostasis is maintained by immunomodulation of the gut mucosa. This is promoted by the interaction of endogenous microbiota with, food and a large variety of vitamins and nutrients, generating a proper intestinal immunity, which in turn maintains an adequate systemic immunity, highlighting the importance of the interaction between the gut mucosa immunity and the systemic immune response (24, 25). In parallel, protein-energy undernutrition is also a systemic condition that causes atrophy of lymphoid tissues, and the thymus is one of the most affected organs, with severe thymocyte loss, particularly CD4^+^ CD8^+^ cells (26–33). The thymus undergoes atrophy, as seen by histology, with changes in the lymphoid and microenvironmental compartments (26, 28). It was observed that in severely malnourished mice and children the thymic intralobular extracellular matrix containing fibronectin, laminin and type IV collagen increased. The enhancement of these extracellular matrix was correlated with the degree of thymocyte depletion in both humans and experimental models (26–29), although a cause-effect relationship has not been determined so far.

**FIGURE 3:**
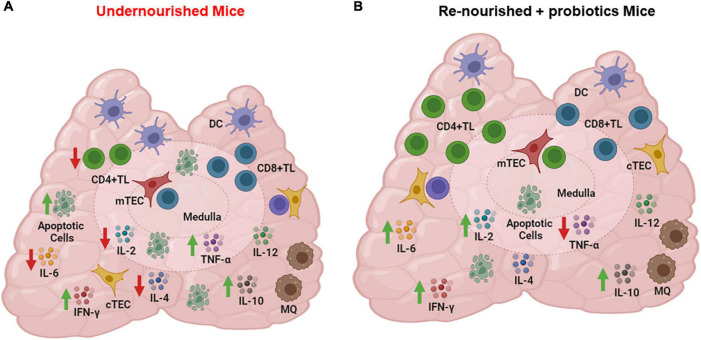
Cytokine producing cells in thymus of undernourished mice. **(A)** Thymic cells from undernourished mice exhibited a significant reduced production of IL-2, IL-6, and IL-4 (red arrows) and a significant increased production of TNF-α, and IL-10 (green arrows). **(B)** Thymic cells from renourished mice fed with probiotic fermented milk showed a significant increase in the production of IFN-γ, IL-2, IL-6, and IL-10 (green arrows) and reduction of TNF-α (red arrow). Cytokine production was detected by indirect immunofluorescence assays. Figure created with BioRender.com, based on data from Maldonado Galdeano et al. (30).

In any case, it is interesting that even in mice developing non-severe undernutrition by food restriction, there is a marked thymic atrophy and cortical thymocyte depletion, due to massive apoptosis, as well as a significant depletion in all the cytokine producing cells assayed (IL-12, IL-4, IL-6, IL-10, TNF-α, IFN-γ), as compared to control animals (30), as summarized in Figure 3.

Finally, although not detailed herein, it is worthy to mention that deficiencies in vitamins, as well as in micronutrients (zinc, for example) also promote thymic atrophy with cortical thymocyte depletion and changes in the microenvironmental compartment (31). Particularly for the zinc, this should be linked to the fact that this element is important in several reactions in the thymus, being not only necessary to thymulin biological functions but also for promoting regeneration of TECs and T-cell reconstitution (22, 34).

## The thymic microenvironment in protein undernutrition and infection

In addition to the lymphoid compartment, the thymic microenvironment is affected in various undernutritional and infectious conditions. Morphological changes in the thymic epithelium from protein-undernourished mice include the decrease in the volume of the epithelial tissue in the cortex and in the medulla of thymic lobules from undernourished mice, as compared to well-nourished control animals (32). By contrast, an increase, of intracytoplasmic accumulations of large, circular, homogeneously electron-dense profiles, rich in free and esterified cholesterol was reported in both in cortical and medullary TEC, of undernourished animals (33). Unfortunately, no data were reported concerning TEC death in this experimental model. In malnourished children the atrophy of the organ is clear with strong diminution of the cortex of the thymic lobules, with concomitant appearance of apoptotic thymocytes, particularly in the CD4^+^ CD8^+^ stage of differentiation.

In *Trypanosoma cruzi*-acutely infected mice, we also demonstrated changes in the expression of medullary and cortical specific TEC markers such as cytokeratins, as compared to controls, together with a general shrinkage of the thymic epithelial network (35). Such phenotypic changes do not prove functional changes of these cells in relation to the interaction with developing thymocytes. However, they did unravel an intrathymic pathology comprising both the cortical and medullary TEC network.

One functional parameter that has been largely evaluated in undernutrition conditions is the thymic hormone production by TEC. It was initially found that protein-undernourished mice exhibited abnormally low levels of circulating thymulin (32, 36), and that such a decrease was also observed in protein-undernourished rats and humans (37). Interestingly, even in human protein undernutrition secondary to *anorexia nervosa*, low thymulin serum levels were reported (38). Furthermore, decreased thymulin serum levels were reported in mice submitted to diets designed to trigger deficiency in zinc, iron, or vitamins (36, 39, 40). At least regarding zinc deficiency, similar results were found in humans (41). Remarkably, in severe infection conditions thymic endocrine function is also affected. We observed in *T. cruzi*-infected mice a transient decrease in the serum levels of the thymic hormone thymulin (31). In human HIV infection we and others showed a consistent and long-term diminution of thymulin secretion, in terms of both serum levels and intrathymic contents of the hormone (39–44).

It is noteworthy that the decrease in thymic hormone serum levels seen in undernutrition is not restricted to thymulin, since it was also reported as regards thymopoietin production. Prenatal undernutrition was significantly associated with reduced thymopoietin production in interaction with the duration of exclusive breast-feeding (45). These findings provide support for the importance of fetal and early infant programming of thymic function, and long-term implications for the immune system, and consequently adult disease risk.

As mentioned above, in addition to the abnormalities seen in thymic epithelial cells, the thymus from undernourished children presents a further microenvironmental alteration, namely, an increase in the deposition of ECM proteins. We studied by histological, ultrastructural and immunohistochemical means, thymuses obtained in necropsies from undernourished children. There is a consistent increase in the intralobular ECM-containing network, which could be ascertained histologically by the dense reticulin staining, and immunohistochemically by the higher contents of fibronectin, laminin, and type IV collagen. Importantly, the enhancement of thymic ECM in undernourished individuals positively correlated with the degree of thymocyte depletion (26). This correlation may represent a cause-effect relationship in which the contact of thymocytes with abnormally high amounts of thymic ECM triggers and/or enhances programmed cell death. However, this notion remains hypothetical, demanding experimental demonstration. In any case, it is noteworthy that developing thymocyte do express integrin-type ECM receptors (21), which makes this hypothesis feasible.

Interestingly, similar changes in thymic ECM were observed in glucocorticoid-hormone treated mice and TEC cultures (22), leading to hypothesis that the enhanced ECM deposition seen in undernutrition may be also related to high levels of serum glucocorticoid hormones. Actually, high glucocorticoid levels are seen in undernutrition (46, 47). Such an alteration was also seen in acute infections, as exemplified by experimental Chagas disease (35, 48). In this infection model, changes in ECM were accompanied by alterations in the migratory response of thymocytes, with abnormal export of CD4^+^CD8^+^ immature thymocytes, some of them having bypassed the normal negative selection process (48–50). Whether similar cell migration abnormalities exist in undernourished subjects, is to be investigated, although peripheral CD4^+^CD8^+^ T cells have been detected in the blood of chagasic patients (51).

Along with the changes in the thymic microenvironment seen in undernutrition conditions, obesity also induces severe alterations in this compartment, as seen in experimental models. One is the db/db mouse that develops a diabetes with obesity, due to a deficit in the expression of leptin receptor, which leads to obesity and type II Diabetes (52). We showed a precocious thymic involution in these animals, with cortical thymocyte depletion as well as changes in the TEC network, as ascertained by ultrastructural changes in these cells and a precocious decrease in the production of the TEC-derived thymulin (53–56). Again, these thymic events may be due to high corticosterone serum levels seen in these mice (57). In any case, these results unravel a thymic alteration in obese animals, which is likely related to an altered adaptive immune response. Whether such changes exist in humans need to be determined. Yet, it is conceivable since during age-dependent thymic atrophy, there is an increase in the numbers of adipose cells in the thymus (58).

## Hormonal imbalance underlines thymic changes in protein undernutrition

Besides the alterations above cited, the impact of protein undernutrition in the thymus is accompanied by a hormonal imbalance in the organ. As mentioned above, decrease in thymic hormone production was first described in the 1970 decade, unraveling reduced levels of thymulin in the serum of children with severe undernutrition, in small for gestational age infants, and in thymuses of children who died in various stages of undernutrition (59, 60). In this case, children with the severe forms (marasmus, kwashiorkor, and marasmic kwashiorkor) presented tiny thymuses that contained very low contents of thymulin (37).

Other than thymic hormones, it has been shown that the circulating levels of corticosterone are increased in protein-undernourished mice, as compared to age-matched controls. High corticosterone levels are known to induce thymic atrophy by the depletion of immature thymocytes, which was also observed in protein-undernourished mice (46). Accordingly, we might predict a dysregulation of glucocorticoid control mechanisms.

The HPA axis encompasses the hypothalamus, with the secretion of the neuropeptide CRH (corticotrophin-releasing hormone), which in turn stimulates the adenopituitary gland to secrete ACTH (adrenocorticotrophic hormone) that enhances glucocorticoid secretion by the cortex of the adrenal glands. Physiologically, such axis is self-regulated by negative feed-back with glucocorticoids being able to negatively control the production of both ACTH and CRH. Yet, it has been demonstrated that maternal food restriction during the perinatal period or during lactation disturbs the activity of the HPA axis at weaning, with pups presenting reduced adrenal, thymus and liver weight, and increased circulating free corticosterone levels (61). Disturbances in the HPA axis during protein deprivation were also reported (62). To determine if corticotropin-releasing hormone (CRH) and glucocorticoids were respectively required for hypophagia and catabolism in undernutrition, CRH-deficient mice were subjected to dietary protein deprivation. Interestingly, these animals did not exhibit increased plasma corticosterone as control individuals. In the same vein, CRH deficiency attenuated body and thymus weight loss induced by the restricted diet, suggesting that those effects were dependent on glucocorticoid regulation.

Mild maternal protein deprivation during lactation in rats affects thymic homeostasis in the young progeny, which presents lower body and thymus weights, significant alterations in CD4/CD8-defined T cell subsets and enhanced expression of leptin receptor ObRb in thymocytes. Although alterations in leptin circulating levels were not observed in this study, an increase in leptin signaling response of thymocytes from protein-deprived rats was described, together with a decreased rate of thymocyte spontaneous apoptosis when compared to controls (63). Interestingly, leptin/leptin receptor-deficient animals exhibit an atrophy of lymphoid tissues, particularly the thymus, and such a defect can be reversed by the reposition of the hormone (64, 65). Actually, complementary studies showed that there is a balance between systemic levels of leptin and glucocorticoids, controlling thymic atrophy. It is thus conceivable that in malnutritional states, the imbalance between the levels of leptin and glucocorticoid hormones could be, at least in part, responsible for the thymocyte depletion and consequent thymic atrophy (31). Yet, further studies are necessary to define if such imbalance is also involved in infection-related thymic atrophy.

## Undernutrition yields a further negative impact of acute infections upon the thymus

It is known that the thymic atrophy induced by undernutrition produces a negative impact in the immune response against infections (31, 66, 67). In this respect and considering that protein-energy undernutrition can be reversed with an appropriate re-nutrition, it might be possible to recover the functional capacity of the thymus, through adequate food intake. Actually, this is definitely plausible since thymopoiesis is a continuous process along with life, although decreasing with aging. This is important particularly in facing infant malnutrition and infection, telling us that we must develop public policies to extinguish food insecurity and hunger on the planet.

As mentioned above, several findings in humans and mice showed that the thymus is also a target for infection (68, 69), as it has been well described for various types of pathogens, including viruses, bacteria, and parasites, such as *T. cruzi*, *Plasmodium* and *Leishmania* (Table 1). In fact, different infectious agents are able to reach and infect the organ. On this regard, we have recently shown that the thymus of BALB/c mice is a target during experimental infection by *Leishmania infantum* (70). The presence of the parasite infecting cells of the thymic microenvironment was observed both in well-nourished animals and in malnourished mice. However, it remains to be determined which cell populations are permissive to the parasite infection. Interestingly, it was possible to observe many more intact amastigotes in the undernourished animals than in the well-nourished ones (70). Recent reports have confirmed, using bioluminescence, the presence of the parasite in the thymus of well-nourished BALB/c mice infected with *L. donovani* (71). These observations demonstrate that the thymus is a target organ during infection by viscerotropic species of *Leishmania*, including *L. infantum* and *L. donovani*. This was further confirmed by the presence of the parasite in the thymus in dogs naturally infected with *L. infantum* (72). In a broader way, several data show that various parasites as well as viruses and even fungi, can be found within the thymus parenchyma. For example, in respect to Chagas disease, previous work had revealed that both epithelial and non-epithelial thymic microenvironmental cells can be infected by *T. cruzi*, as ascertained both *in vitro* and *in vivo* (68).

**TABLE 1:** List of pathogens detected in the thymus.

	Infectious agent	Alterations observed in the thymus	Type of infection (organism)	References
Protozoa parasites	*Leishmania infantum*	Increased cortex:medulla index; altered abundance of extracellular matrix proteins and cell migration-related molecules; altered thymocyte homeostasis	Experimental (Mouse) Natural (Dog)	(69, 70, 72)
	*Leishmania donovani*	Not reported	Experimental (Mouse)	(71)
	*Trypanosoma cruzi*	Atrophy; alteration in extracellular matrix; depletion of DP cells; early export of DN/DP cells; increased expression of cell adhesion and cell migration-related molecules.	Experimental (Mouse)	(15, 73–76)
	*Plasmodium berghei*	Atrophy; histological alterations; increased apoptosis; DP cells depletion, changes in cell migration-related molecules, early release of DN/DP cells	Experimental (Mouse)	(77–79)
	*Toxoplasma gondii*	Atrophy; decreased thymic output; parasite-induced destruction of the thymic epithelium; altered thymic microarchitecture.	Experimental (Mouse)	(80, 81)
Bacteria	*Mycobacterium tuberculosis*	Increased iNOS, IFN-γ and TNF expression	Experimental (Mouse)	(82–84)
	*Mycobacteria*	Not reported	Natural (Human)	(85)
	*Mycobacterium avium*	Atrophy; pathogen-specific immune tolerance	Experimental (Mouse)	(82, 83, 86, 87)
	*Salmonella Typhimurium*	Atrophy; thymocyte apoptosis; depletion of DP cells	Experimental (Mouse)	(88, 89)
	*Francisella tularensis*	Atrophy; depletion of DP cells	Experimental (Mouse)	(90)
Viruses	Influenza virus	Atrophy; Depletion DP cells; Decreased TCR repertoire diversity; Increased apoptosis index	Experimental (Mouse)	(91–93)
	Mouse Hepatitis virus (MHV)	Atrophy	Experimental (Mouse)	(94–96)
	Human Immunodeficiency virus (HIV)	Atrophy; decreased thymic output; depletion of DP, CD4^+^ cells	Natural (Human)	(97–104)
	Zika virus	Cortical atrophy; alteration in extracellular matrix	Natural (Human)	(105, 106)
	Coxsackievirus	Hypertrophy; disruption of T cells export; sjTREC frequencies decreased; depletion of DP cells; altered TEC gene expression	Experimental (Mouse) TEC primary cultures (Human)	(107–115)
	Cytomegalovirus (CMV)	Atrophy; reduced IL-1 secretion	Experimental (Mouse) TEC primary cultures (Human)	(116–119)
	Measles virus	Cortical atrophy; Depletion DP cells	Experimental (Mouse)	(120–122)
	Porcine reproductive and respiratory syndrome virus (PRRSV)	Atrophy; decreased thymic cortex; thymocyte apoptosis; thymic epithelial cell autophagy.	Experimental (Piglets)	(123, 124)
	Lymphocytic choriomeningitis virus (LCMV)	Atrophy; severe thymocyte depletion; impaired thymic negative selection; escape of self-reactive T cells; pathogen-specific immune tolerance	Experimental (Mouse)	(125, 126)
	Murine eucemia virus (MLV)	Atrophy; thymocyte apoptosis	Experimental (Mouse)	(127, 128)
	Herpesvirus: Murine roseolovirus (MRV) Mouse thymic virus (MTLV)	Atrophy, thymic necrosis; T cell depletion	Experimental (Mouse)	(129, 130)
	Poliovirus	Not reported	Natural (Human)	(131)
	Epstein-Barr virus	Increased TLR7 and TLR9 expression in thymic epithelium	Natural (Human)	(132–134)
Fungi	*Paracoccidioides brasiliensis*	Atrophy; histological disorganization; depletion of TEC, DP, CD4^+^, and CD8^+^ cells; defects in selection processes	Experimental (Mouse)	(135–137)
	*Cryptococcus neoformans*	Changes in thymic architecture	Experimental (Rat)	(138, 139)

As previously mentioned, immunosuppression resulting from protein-energy undernutrition severely impacts primary and secondary lymphoid organs, involved in the response to pathogens, contributing to mortality and morbidity, especially in children (10). In addition, systemic hormonal and metabolic dysfunctions, as well as changes in intestinal barrier function and intestinal microbiota, caused by undernutrition, dramatically increase the susceptibility of individuals to infections, as seen in many different examples, such as visceral leishmaniasis (VL), influenza, dengue, Zika and tuberculosis, among others (10, 67, 140–151). In children aged less than 5 years, undernutrition is an underlying cause of 61% of deaths from diarrhea, 57% of deaths from malaria, 52% of deaths from pneumonia and 45% of deaths from measles (152). Furthermore, it has been estimated that the COVID-19 pandemic will increase childhood mortality from wasting by more than 20% (153). Indeed, recent report already described increased rates of fatal COVID-19 in areas with elevated burden of undernutrition (154).

Another example of the deleterious role of acute infections in undernourished subjects it the study on predictors of mortality in adult patients with influenza infection in Switzerland. This analysis was conducted during four influenza seasons and identified undernutrition as a strong predictor of mortality among those patients (155). Experimental model of protein-energy undernutrition and influenza A virus infection showed that mice fed low-protein diet (2% protein content) exhibited more severe disease than mice fed a control protein diet (18% protein content). Undernourished animals presented higher and sustained virus titers in the lungs, trafficking of inflammatory cells to the lung tissue, and higher virus-induced mortality, when compared to what was seen in control mice. Interestingly, undernourished-infected mice fed with control diet improved virus clearance, as well as recovered protective immunity to viral challenge (67).

It has also been suggested that maternal protein undernutrition increases susceptibility to Zika virus infection, which causes the Congenital Zika Syndrome in children. This syndrome is characterized by multiple neurological, muscular and immune disturbances, secondary to infection in pregnant mothers. In neurological terms, the children may present severe microcephaly, decreased brain tissue with a specific pattern of brain damage, including subcortical calcifications, damage to the back of the eye, with macular scarring and focal retinal pigmentary mottling, congenital contractures, such as clubfoot or arthrogryposis and Hypertonia restricting body movement soon after birth (156). From an experimental perspective, it was showed that pregnant mice subjected to protein undernutrition and infected with Zika virus presented severe alterations of placental structure and embryonic body growth, with offspring displaying a reduction in neurogenesis and postnatal brain size as well as alterations in the expression of genes required for brain development (142). Still regarding arboviruses, experimental models of Dengue virus (DENV) infection have shown that, compared with well-nourished animals, mice fed low protein content diet (5% protein) had a significant reduction in the level of platelets, increased spleen pathology and higher viral titers in the spleen following infection (143). However, studies regarding the association between the nutritional status and dengue infection in humans are controversial; some studies reporting higher risk of dengue shock syndrome or dengue hemorrhagic fever in undernourished children, whereas other studies could not observe these associations (157, 158). Consequently, more data are needed to clarify the influence of nutritional status on dengue infection outcome.

Another controversial association is that observed between malaria and undernutrition. Data from different analyses present conflicting conclusions regarding the potentially protective or exacerbating effects that undernutrition has on *Plasmodium spp*. infection (159). Because in endemic regions there is a common seasonality of malaria and undernutrition (160), it is difficult to clearly point if one is cause or consequence of the other. Some studies suggested that undernutrition increases the susceptibility of children to infection and/or impairs the individual’s capability to recover from infection (161–163). It has been observed that malnourished children have decreased specific anti-*Plasmodium* antibody titers when compared to well-nourished children (164). On the other hand, data related to the risk of malaria infection in children with chronic undernutrition revealed a protective effect of undernutrition against infection (165, 166). Several studies in experimental models indicate that animals submitted to a low-protein diet had reduced parasitemia when compared to controls; however, the immune response was also suppressed, and the animals were unable to clear the infection (167, 168). Interestingly, the immunosuppression observed during the deficit in protein consumption makes parasitized animals protected from experimental cerebral malaria (169, 170), a severe form of malaria directly associated with an exacerbated inflammatory response. Notably, it was observed that a brief restriction of food intake prevents neuropathology in an experimental cerebral malaria murine model with *P. berghei* ANKA (170). One hypothesis that should be investigated is that undernutrition would induce lower inflammatory and adaptive immune response that would partially undermine the autoimmune environment seen in both human and experimental malaria (171).

In general, infection or undernutrition induces similar alterations to lymphoid organs. Independently, undernutrition or acute infectious diseases result in thymic atrophy with drastic reduction of immature CD4^+^CD8^+^ double positive (DP) T cells due to increased apoptosis and premature egress of immature thymocytes (29, 36, 68, 77, 92, 172–174). Intrathymic chemokines and extracellular matrix (ECM) components are also altered during pathological conditions of infection or undernutrition. The thymus of *T. cruzi*-infected mice exhibits increased fibronectin and chemokine deposition, and increased abundance of thymic ECM proteins such as fibronectin, laminin, and type IV collagen has been observed in undernourished children (26, 49). Thymus atrophy with thymocyte depletion, increased intra and inter-lobular connective tissue, and decreased cortico-medullary limits have been also observed in undernourished children (175–177). Indeed, a smaller thymus is a risk factor for mortality and is predictive of decreased immune competence (178). As mentioned above, one of the mechanisms underlining these effects on thymocytes is be related to the hormonal imbalance between the rise in glucocorticoids and the decrease in leptin (50). Yet, other hormonal circuits may be involved as seen in experimental acute Chagas disease, in which prolactin partially reverts the thymic atrophy and the corticosteroid levels (179). Should we emphasize however, that other non-hormone-mediated mechanisms may be involved, and should be investigated.

## Consequences of undernutrition and *Leishmania* infection upon the thymus: Cellular and proteomic approaches

Visceral leishmaniasis (VL) is a neglected disease that frequently afflicts children and malnourished populations in tropical and subtropical regions of the world. The disease is caused by *Leishmania infantum* or *L. donovani* parasites, which infect the spleen, liver, bone marrow, and lymph nodes, causing fever, hepatosplenomegaly, and loss of weight. If not properly treated, VL can be fatal (180, 181).

Seminal prospective studies in children who acquired VL revealed that those who had a precondition of moderate to severe undernutrition before infection had 8.7 times greater risk of having classic and severe forms of the disease, whereas well-nourished children did not progress beyond subclinical infections (140). More recent epidemiological surveys revealed that in cases of VL in adults who died, 32.7% of patients had undernutrition as the main comorbidity (182). In Northwest Ethiopia it was observed that roughly 85% children aged under 5 years and 95.5% adults with VL were undernourished (183, 184).

Studies of the relationship between VL and undernutrition in experimental murine models established a murine scale of undernutrition based on weight for age in analogy to the anthropometric classification of human undernutrition. Using this model it was found that undernutrition led to a failure in the barrier function of the lymph nodes in mice infected with *L. donovani* and, consequently, anticipated the visceralization of the parasite (185), likely due to the reduction of phagocytic cells in the lymph nodes of undernourished animals (186). A reduction in the number of skin-resident dendritic cells (DCs) drained to satellite lymph nodes was also observed, as well as a deregulation in the expression of chemokines and corresponding receptors, involved in the migration of DCs to the lymph nodes (187).

Also, in an undernourished murine model of infection with *L. infantum*, we observed a drastic reduction in cellularity and changes in the microarchitecture of the spleen and thymus of infected animals, as well as changes in the subpopulations of thymocytes and splenocytes, which were aggravated when the infection was preceded by undernutrition (69, 70, 188, 189). Indeed, undernourished-infected mice exhibited a significant reduction in the cortex:medulla ratio (69).

Aiming at understanding the effects of protein undernutrition in the course of infection and immune response to *L. infantum*, we established a model of protein undernutrition and infection with *L. infantum*, which has allowed us to describe cellular and molecular alterations in the thymus, spleen and gut of mice and how they affect the response to the parasite (69, 70, 188–190), using BALB/c mice fed control (14% protein) or low protein (4% protein) isocaloric diets further infected with *L. infantum* infection. Undernourished-infected mice exhibited a significant reduction of body and thymus weight, showed a significant decrease in CD4^+^CD8^+^ (DP) thymocytes, and remarkable alterations in total single positive subpopulations (CD4^+^ and CD8^+^) of the organ (69, 70, 188). Those changes were accompanied by reduced systemic levels of leptin and IGF1 and increased corticosterone, all of which have been associated with thymocyte depletion in undernourished individuals (66). Notably, those defects were induced independently by each condition (undernutrition or parasite infection), but the synergy of both exacerbated the observed alterations, accelerating the pathological events seen during infection (69, 70, 188). We further observed a decreased intrathymic abundance of CCL5, CXCL12, CXCL9, and CXCL10 as well as IGF1 in undernourished-infected animals, suggesting altered migration of developing T cells. Nevertheless, in this combined condition thymocytes were able to migrate *ex vivo* in response to chemotactic stimuli, suggesting that undernutrition may compromise the production of soluble factors inside the thymic microenvironment, altering *in vivo* thymocyte migration, rather than migratory capability of T cells *per se* (70).

As mentioned above, the successful production of mature T cells depends on the constant migration of differentiating thymocytes through the thymic microenvironment, and such migration is a process controlled by complex interactions between cell surface molecules, extracellular matrix (ECM) proteins, cytokines, chemokines and hormones (21, 191, 192). We conducted a histopathological study of the tissue organization and using a quantitative mass spectrometry-based proteomics approach, to measure the protein abundance within the thymic interstitial space in undernourished BALB/c mice infected with *L. infantum* (69). Undernourished-infected animals exhibited a significant reduction of the thymic cortical-medullary ratio and altered the abundance of proteins secreted in the thymic interstitial fluid. Proteomic analyzes revealed that these alterations were accompanied by a significant change in the abundance of soluble factors that are secreted via exosomes into the thymic microenvironment, suggestive of defects in the intrathymic molecular communication mediated by these microvesicles in undernourished animals (69).

**FIGURE 4:**
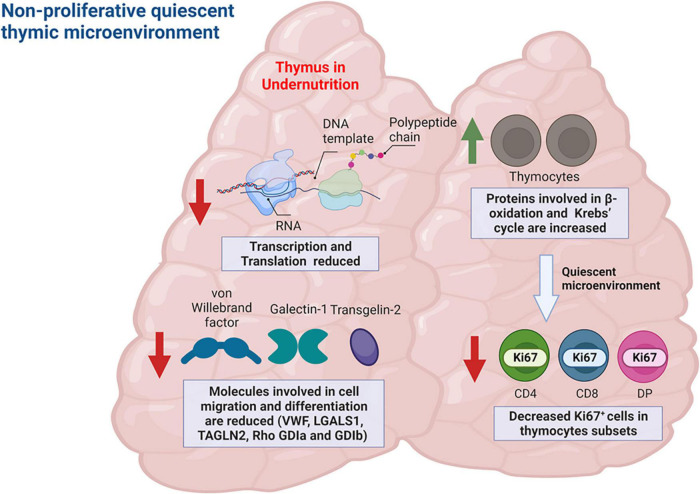
Quantitative proteomics analysis of thymic interstitial fluid of undernourished-infected mice reveals a non-proliferative quiescent thymic microenvironment. Examples of proteins with differential abundance in the undernourished-infected mice relative to the control (well-nourished) mice are represented in the figure. Proteins involved in transcription and translation as well as proteins involved in cell migration and differentiation were reduced in undernourished mice (red arrows). Conversely, proteins involved in fatty acid beta-oxidation and Krebs’ cycle were increased in those animals (green arrow), compatible with a quiescent, non-proliferative metabolic profile. Such profile was corroborated by a decreased percentage of proliferating thymocyte subsets expressing Ki67. Figure created with BioRender.com, based on data from Losada-Barragán et al. (69).

Also, early changes in protein abundance were observed in infected animals, and those alterations were exacerbated or annulated when animals were previously undernourished, reinforcing the deleterious role of undernutrition in the response to infection and revealing an unknown role of the thymus during VL (69). Functional analysis of proteomics data showed that molecules involved in cell migration and differentiation such as galectin-1, von Willebrand factor, Rho GDP-dissociation inhibitor 1 and 2, differentiation factor 1 and transgelin-2 were significantly reduced in undernourished-infected mice (69). In addition, we observed an increase in the amounts of proteins involved in β-oxidation of fatty acids as well as in those involved in Krebs’ cycle, both of which suggest a non-proliferative quiescent thymic microenvironment (Figure 4), which in fact was corroborated by the decreased detection of Ki67 proliferation marker in thymocyte subpopulations (69). Together these data compose a scenario where structural and soluble protein factors of the thymic microenvironment are altered by *L. infantum* infection and worsened by a precedent undernutrition condition.

As mentioned above, we detected parasites in the thymus of both well-nourished and undernourished animals infected with *L. infantum*, but amastigote nests were only observed in mice fed protein restricted diet (70). Furthermore, using qPCR, we observed a significantly higher parasite load in the thymus of undernourished animals when compared to the control mice (193).

Interestingly, T cells generated in the thymus previously infected by *Mycobacterium avium* are tolerant to the pathogen in the periphery (86). In this line, the persistence of infection and antigenic peptides in the thymus may favor the appearance of pathogen-tolerant T cells, thus contributing to its persistence in other tissues (194). In a similar way, the persistence of *L. infantum* in the thymus of undernourished animals could favor the emergence of *Leishmania*-tolerant T cells, impairing the resolution of the infection in the periphery, favoring parasite persistence in the spleen. Such reasoning can also apply for different intrathymic infections.

## Probiotics: Potential immunotherapeutic tools for restoring undernutrition related thymic dysfunctions

A large reservoir of microorganisms is found in the gut, reaching a bacterial community that is 10 times more than the number of human eukaryotic cells, coexisting in a symbiotic relationship (195). The role of these microorganisms in the gut physiology and maintaining intestinal homeostasis is indisputable. They participate in the breakdown of food, nutrient absorption, synthesize vitamins, protect us against pathogens, act on the neurodevelopment, and participate in the development and regulation of the immune system (196, 197). The microbiota composition differs from one individual to another, being in direct relation to age, diversity of food consumed, lifestyle, ethnicity, environmental factors and following the use of medicines such as steroids, antibiotics, etc. (198, 199). Among the millions of microorganisms in the human digestive tract, probiotics are found. They can influence the intestinal immune system by several mechanisms including change in the microbiota composition and its function, as well as improvement of the intestinal epithelial barrier (200, 201).

The mechanisms involved in the remote effects of probiotic are poorly understood. Yet, they can balance the microbiota and modulate the expression of pro-inflammatory cytokines. Such an effect occurs through multiple mechanisms, including the modulation and stimulation on MAPK (mitogen-activated protein kinase) pathways, as well as upon the NF-kB transcription factor, especially by inhibiting IkB phosphorylation, thus hindering the transfer of NF-kB (202).

Much less is known about the putative action of probiotics upon the thymus. In a mouse model of non-severe protein undernutrition, we found that the administration of a probiotic fermented milk as a re-nutrition supplementation did improve the thymic microarchitecture, recovering the corticomedullary differentiation in the thymic lobules, in conjunction with a decrease in thymocyte apoptosis. All the structural changes of thymus were accompanied by changes at the functional level, with increase in the numbers of mature and immature thymocytes and enhancement of cytokine production (203).

**FIGURE 5:**
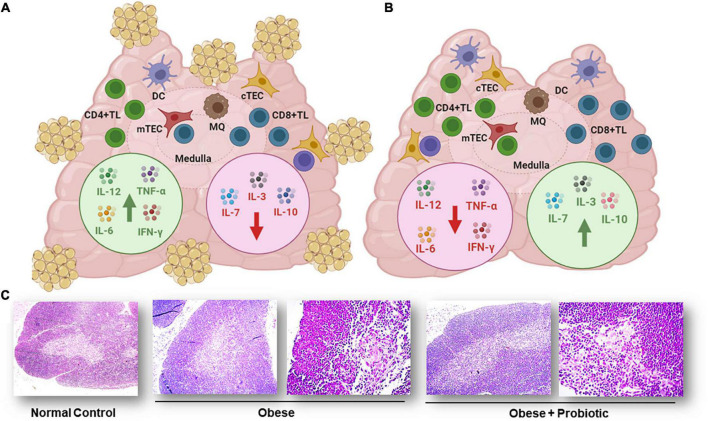
Cytokine production in thymus of obese mouse model. **(A)** Thymus from obese animals showed adipose deposits covering the tissue, a significant decrease in thymocytes, increased levels of IL-12, IL-6, TNF-α, and IFN-γ (green arrow) and decreased production of IL-3, IL-7, and IL-10 (red arrow). **(B)** Thymus from obese mice fed probiotic bacteria *L. casei* CRL 431 did not exhibit visible adipocytes, recovered thymocyte numbers, showed decreased levels of IL-12, IL-6, TNF-α, and IFN-γ (red arrow), and exhibited increased levels of IL-3, IL-7, and IL-10 (green arrow). Cytokines were measured by ELISA in the supernatants of cultured thymocytes. Figure created with BioRender.com, based on data from Balcells et al. (206). **(C)** Micrographs of thymus sections from normal control and obese mice. Tissue sections from normal control (100×), obese control (100× and 400×) and obese mice feed with probiotic bacteria (100× and 400×) stained with hematoxylin and eosin. Figure modified from Balcells et al. (206).

In a second vein, studies in obese humans and in obese mice, showed a relation between obesity and involution of the thymus gland revealing the important role of nutrients intake on the anatomy and functionality of the thymus (65, 203, 204). The use of a probiotic yogurt as dietary supplementation resulted not only in the control of body weight, serum biochemical parameters, but also in the recovery of the histological structure and thymus weight in obese animals (205). This was confirmed by recent data showing that a probiotic strain *L. casei* CRL 431 administered in the drinking water to a high-fat diet consuming obese mice, improved the cellularity and functionality of the thymus, with an increase in IL-7 and IL-3 production as seen in Figure 5 (206). These two cytokines are involved in normal intrathymic T-cell development; IL-7 is secreted by TEC and stimulates survival and expansion of the immature thymocytes and increase thymocyte numbers (207) and IL-3, also produced by TECs, is a hematopoietic growth factor that promotes myeloid proliferation (208).

## Concluding remarks and perspectives

As undernutrition remains a major issue in global health, studies on the pathophysiological aspects of this affection, associated or not with infection are yet important, particularly the mechanisms governing dysregulation of the immune response. In this respect, the immunosuppression resulting from protein-energy undernutrition severely impacts primary and secondary lymphoid organs involved in the response to a give pathogen. The thymus is particularly compromised by both undernutrition and infection, with a consistent cortical thymocyte depletion and important changes in thymic epithelial cells. Moreover, undernutrition yields a negative impact upon the thymus in acute infections, frequently with the intrathymic detection of pathogens or their antigens.

**FIGURE 6:**
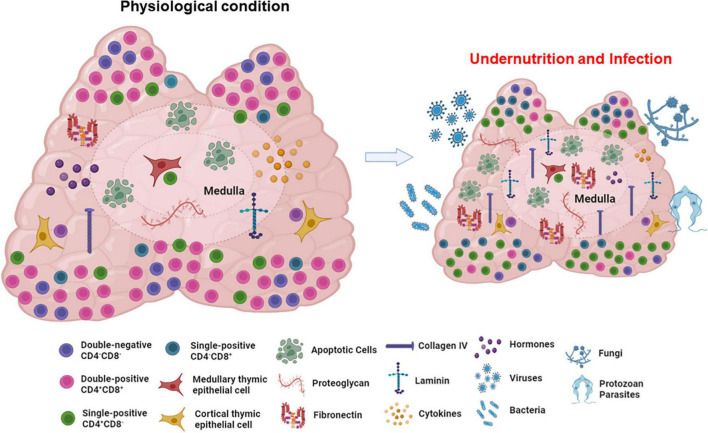
Effects of undernutrition upon the thymic lymphoid and microenvironmental compartments. The scheme clearly shows the atrophy of the thymus in undernutrition conditions, affecting both developing thymocytes and microenvironmental elements of the organ. Importantly, undernourished thymuses are more susceptible to infections and thymic changes are still more pronounced. Figure created with BioRender.com.

The impairment of the thymic cortical area due to undernutrition during acute infections may alter physiological processes crucial for T cell development, such as (i) intrathymic selection of the T cell repertoire (209), (ii) thymocyte proliferation, (iii) adequate migration of developing thymocyte, as well as (iv) changes in the microenvironmental component of the organ, and (v) intrathymic and systemic hormonal circuits (Figure 6), all of which having a deleterious impact in the adaptive immune responses in the periphery. As intrathymic T cell development is a continuous process, it exhibits a certain degree of plasticity, rendering possible the partial (or even total) restoration to normal steady state after a given deleterious stimulus has stopped. Accordingly, it seems plausible that those alterations can be at least partially reverted by dietary interventions, rescuing proper immune responses to infection. However, this remains to be investigated. Addressing whether nutritional rehabilitation combining balanced macronutrients, micronutrients and probiotics can recover the structural and cell differentiation damage caused by undernutrition in the thymus, would allow proposing nutrition-based interventions that would positively impact both the nutritional status of the individuals and their ability to respond adequately to infections.

## Author contributions

WS and PC conceived, wrote, reviewed, and edited the manuscript. JD, CM-G, GP, and DM-d-C wrote the manuscript. All authors contributed to the article and approved the submitted version.
